# H_2_S Donors with Cytoprotective Effects in Models of MI/R Injury and Chemotherapy-Induced Cardiotoxicity

**DOI:** 10.3390/antiox12030650

**Published:** 2023-03-05

**Authors:** Qiwei Hu, John C. Lukesh

**Affiliations:** Department of Chemistry, Wake Forest University, Wake Downtown Campus, 455 Vine Street, Winston-Salem, NC 27101, USA

**Keywords:** hydrogen sulfide, H_2_S donors, cardioprotection, MI/R injury, chemotherapy-induced cardiotoxicity, H_2_S codrugs

## Abstract

Hydrogen sulfide (H_2_S) is an endogenous signaling molecule that greatly influences several important (patho)physiological processes related to cardiovascular health and disease, including vasodilation, angiogenesis, inflammation, and cellular redox homeostasis. Consequently, H_2_S supplementation is an emerging area of interest, especially for the treatment of cardiovascular-related diseases. To fully unlock the medicinal properties of hydrogen sulfide, however, the development and refinement of H_2_S releasing compounds (or donors) are required to augment its bioavailability and to better mimic its natural enzymatic production. Categorizing donors by the biological stimulus that triggers their H_2_S release, this review highlights the fundamental chemistry and releasing mechanisms of a range of H_2_S donors that have exhibited promising protective effects in models of myocardial ischemia-reperfusion (MI/R) injury and cancer chemotherapy-induced cardiotoxicity, specifically. Thus, in addition to serving as important investigative tools that further advance our knowledge and understanding of H_2_S chemical biology, the compounds highlighted in this review have the potential to serve as vital therapeutic agents for the treatment (or prevention) of various cardiomyopathies.

## 1. Introduction

Hydrogen sulfide (H_2_S) is a malodorous, toxic, and flammable gas that was once disregarded as a mere environmental and industrial pollutant [1,2,3]. Landmark studies near the turn of the 20th century [4,5,6], however, revealed that H_2_S is also a biologically active gas that is expressed in mammalian systems, primarily via the enzymatic metabolism of cysteine and homocysteine [7]. From these reports, a paradigm shift ensued, and today H_2_S is regarded as the third gasotransmitter, alongside nitric oxide (NO) and carbon monoxide (CO) [8,9,10,11].

H_2_S is soluble in water (~80 mM at 37 °C [12]) and exhibits weak acidity that gives rise to an equilibrium between its diprotic (H_2_S) and hydrosulfide (HS^−^) forms in an aqueous environment. With a p*K*_a1_ of 6.98 [12], its HS^−^ form dominates at physiological pH and begets its high reactivity and strong nucleophilic character under biologically relevant conditions.

In its diprotic form, its lipophilicity, low molecular weight, and gaseous nature enable H_2_S to easily traverse the lipid bilayer, allowing it to act on intracellular targets that mediate numerous physiological and pathophysiological processes within the human body [13,14,15,16]. Its proven ability to reduce oxidative stress and inflammation [17,18,19], induce vasodilation [6], and promote angiogenesis [20] underscores the positive influence of H_2_S on the cardiovascular system, specifically. Not surprisingly, small molecule donors that improve the exogenous delivery and bioavailability of H_2_S are currently being investigated with great enthusiasm as potential cardioprotective agents [21].

This review will summarize the structure, reactivity, and mode of delivery for H_2_S donors that have displayed promising cardioprotective effects in myocardial ischemia-reperfusion (MI/R) injury and cancer chemotherapeutic-induced cardiotoxicity models, in particular. Thus, the compounds reported on herein not only represent important investigative tools for probing the chemical biology of hydrogen sulfide but may also serve to unlock its vast therapeutic potential for the treatment of cardiovascular-related diseases.

## 2. H_2_S Biosynthesis and Metabolism

In mammals, both enzymatic and nonenzymatic pathways are involved in H_2_S biosynthesis, with the former being the principal route towards its formation. The use of enzymes provides strict spatiotemporal control over the production of H_2_S, resulting in concentration variances in specific tissues and cellular compartments, and in response to certain physiological and pathophysiological events. The three enzymes primarily responsible for H_2_S biosynthesis are cystathionine β-synthase (CBS) [22], cystathionine γ-lyase (CSE) [23], and 3-mercaptopyruvate sulfurtransferase (3-MST) [24].

**Figure 1:**
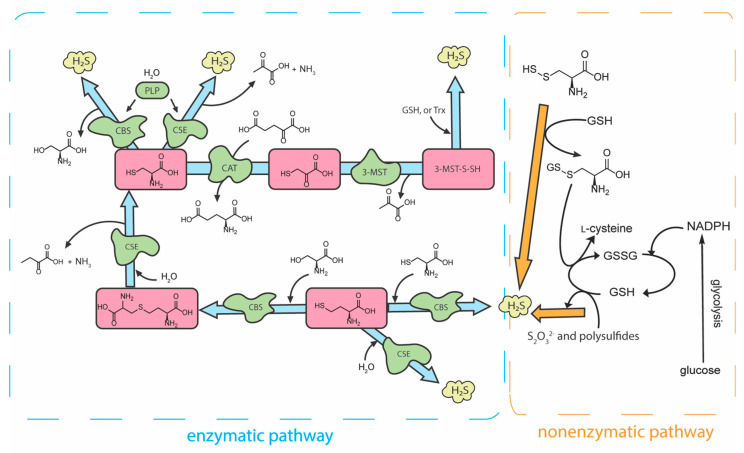
Enzymatic and nonenzymatic production of H_2_S in mammalian systems. PLP: pyridoxal 5′-phosphate; CBS: cystathionine β-synthase; CSE: cystathionine γ-lyase; CAT: cysteine aminotransferase; 3-MST: 3-mercaptopyruvate sulfurtransferase; NADPH: nicotinamide adenine dinucleotide phosphate.

CBS and CSE are ubiquitous enzymes of the transsulfuration pathway that facilitate the conversion of homocysteine to cysteine via the intermediate cystathionine (Figure 1) [25,26]. Both are pyridoxal 5′-phosphate (PLP)-dependent enzymes that are primarily located in the cytosol and generate H_2_S via the direct desulfhydration of cysteine and homocysteine. In addition to being primarily responsible for H_2_S biosynthesis in the brain and central nervous system, CBS is amply expressed in the ileum, kidneys, liver, and uterus [5,27,28]. CSE, on the other hand, exhibits low expression levels in the central nervous system but is the principal H_2_S-producing enzyme of the cardiovascular system [29].

Unlike CBS and CSE, 3-MST is a PLP-independent enzyme that is chiefly expressed in mitochondria and produces H_2_S from the indirect desulfhydration of cysteine [30]. As depicted in Figure 1, in this pathway, cysteine must first be transformed into 3-mercaptopyruvate (3MP) via the enzyme cysteine aminotransferase (CAT). Then, using 3MP as a substrate, 3-MST transfers a sulfur atom onto itself forming a hydropersulfide (3-MST-SSH). In the presence of reductants, 3MST-SSH is reduced, releasing H_2_S in the process.

In addition to the enzymatic routes outlined above, nonenzymatic pathways also contribute to the endogenous production of H_2_S in mammals. In general, sulfane sulfur and other reactive sulfur species (RSS), including hydropersulfides (RSSH), polysulfides (RSS_n_R), and thiosulfate (S_2_O_3_^2−^), serve as effective H_2_S precursors in the presence of glutathione and other reductants (Figure 1) [31,32,33]. To this end, processes that increase the production of nicotinamide adenine dinucleotide phosphate (NADPH), which facilitates the recycling of oxidized glutathione back to its reduced form, have been shown to enhance this nonenzymatic pathway and promote H_2_S biosynthesis [8].

While less is known about the metabolism and removal of H_2_S from mammalian systems, the primary pathways are believed to involve mitochondrial oxidation [34,35], cytosolic methylation [36], hemoglobin and metalloprotein binding [37], expiration via the lungs [38], and its storage in proteins as bound sulfane sulfur [39]. The majority of H_2_S is ultimately excreted via the kidneys in the form of sulfate (SO_4_^2−^) [40]. This oxidation of H_2_S occurs in mitochondria and is facilitated by the enzymes sulfide quinone reductase (SQR) and rhodanese. This metabolic process also accentuates the biological activity of H_2_S and its ability to stimulate oxidative phosphorylation and ATP production through its donation of electrons to the mitochondrial electron transport chain through SQR and mitochondrial complex II [41,42].

## 3. H_2_S Bioactivity and Its Attenuation of Myocardial Ischemia-Reperfusion Injury

**Figure 2:**
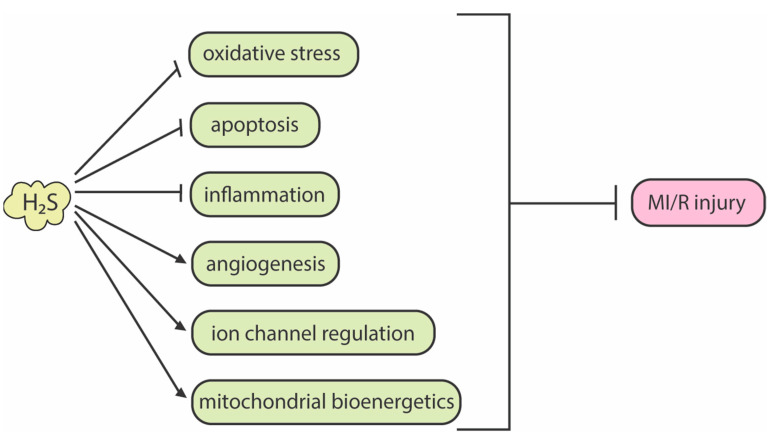
Molecular mechanisms that underscore the cardioprotective effects of H_2_S, including its ability to combat MI/R injury.

In addition to serving as a mitochondrial protectant and stimulator of mitochondrial bioenergetics, endogenous H_2_S has been shown to play a key role in several other physiological and pathophysiological processes [14,15,16,43,44,45,46,47]. The cardiovascular system, in particular, appears to be positively influenced by H_2_S given its involvement in vasodilation and blood pressure regulation [6,48]; its antioxidative [19], anti-inflammatory [17,49], and cytoprotective properties [50,51]; and its ability to promote angiogenesis [20]. Additionally, recent evidence suggests that the co-release of H_2_S (via the transsulfuration pathway) and adenosine (via the methionine cycle) may protect the myocardium from injury [52,53]. For these reasons it is theorized that the exogenous delivery of H_2_S may hold therapeutic value for the prevention and treatment of various cardiovascular-related diseases [54,55], including myocardial ischemia-reperfusion (MI/R) injury [56,57,58,59,60,61,62] (Figure 2).

Myocardial ischemia occurs when blood flow to the heart is restricted due to the buildup of plaque in a coronary artery. If left unchecked, this may lead to myocardial infarction, or heart attack, which is the leading cause of death worldwide [63,64]. To repair myocardial structural damage and prevent ischemic progression, reperfusion therapy is typically employed. This rapid return of blood to ischemic tissue, however, often leads to (MI/R) injury caused by inflammation and oxidative damage [65,66]. Increased levels of reactive oxygen species (ROS), coupled with an overwhelmed antioxidant defense, play a major role in reperfusion injury and can exacerbate cardiac damage that occurs during ischemia [67].

Intracellular calcium overload, a hallmark of reperfusion injury, stimulates the translocation of CSE from the cytosol to mitochondria, which elevates the production of H_2_S within that subcellular space [68]. This innate response of the human body is produced in an effort to preserve mitochondrial function and protect the myocardium from oxidative damage, highlighting the potential for therapeutic intervention with H_2_S delivery. Indeed, recent studies have highlighted the protective effects of exogenous hydrogen sulfide during MI/R. One of the earliest examples in vitro was a study conducted by Johansen and co-workers [69]. Using an isolated perfused heart assay with rats, preconditioning with 1 μM NaHS (an H_2_S equivalent in buffer) 10 min prior to coronary occlusion and up until 10 min post reperfusion, they observed a 20% reduction in infarct size. Pretreatment with Glibenclamide (K_ATP_ blocker) nullified the effect of exogenous H_2_S, which supports its involvement in K_ATP_ channel opening as a primary mechanism of alleviation. Later studies have shown that H_2_S promotes the persulfidation (protein-SSH) of Cys43 of the K_ATP_ protein, resulting in channel opening, an influx of K^+^, and vascular smooth muscle relaxation.

Additionally, sulfide salts have been used to demonstrate the protective effects of H_2_S against MI/R injury in vivo. In an early study by Sivarajah et al. [70], mice were exposed to 25 min of regional myocardial ischemia and 2 h of subsequent reperfusion. When NaHS (3 mg/kg) was delivered 15 min prior to ischemia, a 26% reduction in infarct size was reported in comparison to the vehicle control. Subsequently, Elrod and co-workers investigated the impact of exogenous H_2_S being delivered at the time of reperfusion rather than prior to the ischemic event [71]. In their study, mice were subjected to 30 min of left coronary artery ischemia followed by a 24 h period of reperfusion in the presence of Na_2_S (50 μg/kg). Remarkably, they observed a 72% reduction in infarct size under these conditions.

While sulfide salts, such as NaHS and Na_2_S, serve as convenient H_2_S precursors, their addition to buffered solutions results in a rapid surge in H_2_S concentration, followed by a swift decline due to the instability and transient nature of hydrogen sulfide [72]. Moreover, these characteristics poorly mimic the slow and steady enzymatic production of H_2_S, which often leads to adverse side effects when sulfide salts are employed. For these reasons, small molecule donors designed to release H_2_S in a controlled fashion, and under biologically relevant conditions, have been sought to harness the medicinal properties of H_2_S [73,74,75,76,77].

In the ensuing section, we will highlight examples of small molecule donors that better mimic the natural biosynthesis of H_2_S and exhibit promising cardioprotective effects, especially against myocardial ischemia-reperfusion injury.

## 4. H_2_S Donors That Protect against Myocardial Ischemia-Reperfusion Injury

Hydrogen sulfide donors with success at protecting against MI/R injury are highlighted in Table 1 and arranged by their mechanism for H_2_S release. In this section, the H_2_S releasing mechanism of each donor will be detailed, and their resulting therapeutic effects in various MI/R injury models will be summarized.

### 4.1. Hydrolysis-Triggered Donors

**Figure 3:**
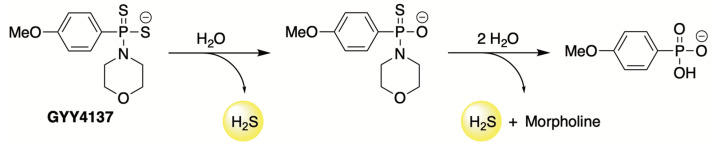
Hydrolysis-triggered H_2_S release from GYY4137.

Morpholin-4-ium 4-methoxyphenyl (morpholino) phosphinodithioate (GYY4137) is the first and most-researched H_2_S donor ever developed [78,79]. It was accessed by treating Lawesson’s reagent with morpholine to impart high water solubility (~30 mg/mL at pH 7.4), which facilitates its use in biological studies. The proposed H_2_S releasing mechanism for GYY4137 is depicted in Figure 3. From detailed mechanistic work carried out by Alexander and co-workers [80], a two-step hydrolysis was put forth, which ultimately yields an arylphosphonate and 2 equiv of H_2_S. The second hydrolysis step, however, was deemed to be too slow to be responsible for any of its observed biological activity, suggesting that GYY4137 primarily undergoes a single hydrolytic P–S bond cleavage event in water to release 1 equiv of H_2_S.

In stark contrast to sulfide salts, GYY4137 is recognized for its ability to provide the slow and continuous release of H_2_S for up to a week after its introduction to water. In its first reported study, GYY4137 was shown to relax rat aortic rings due to its activation of vascular smooth muscle K_ATP_ channels [78]. Moreover, unlike sulfide salts whose effects were brief, GYY4137 was found to be a far more potent vasorelaxant, presumably due to its sustained release of H_2_S and extended interaction with aortic rings. Perhaps not surprisingly, GYY4137 has also exhibited protective effects against MI/R injury [81,82,83]. Beyond its activation of vascular smooth muscle K_ATP_ channels [78,84], additional mechanisms have been invoked which include the ability of GYY4137 to attenuate oxidative stress and apoptosis through increased Bcl-2 expression and its activation of the Nrf2 signaling pathway [81,82].

**Figure 4:**
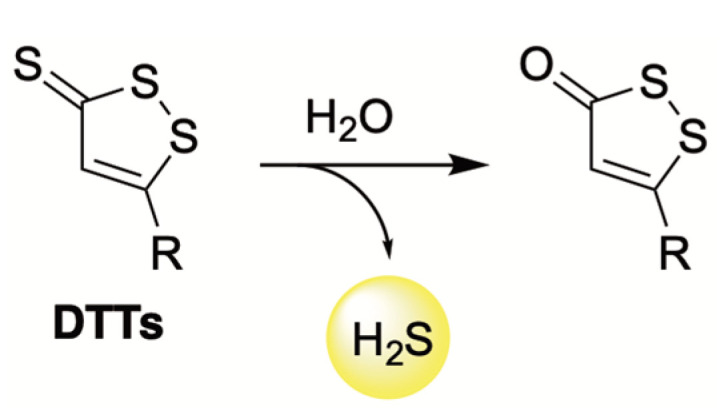
Hydrolysis-triggered H_2_S release from DTTs.

Aside from GYY4137, 1,2-dithiole-3-thiones (DTTs) represent another important H_2_S donating scaffold that operates via chemical hydrolysis (Figure 4) [85]. Although detailed mechanistic studies have yet to be carried out, conventional wisdom suggests that, in water, DTTs are converted into their corresponding 1,2-dithiole-3-one structure with the concurrent liberation of H_2_S.

**Figure 5:**
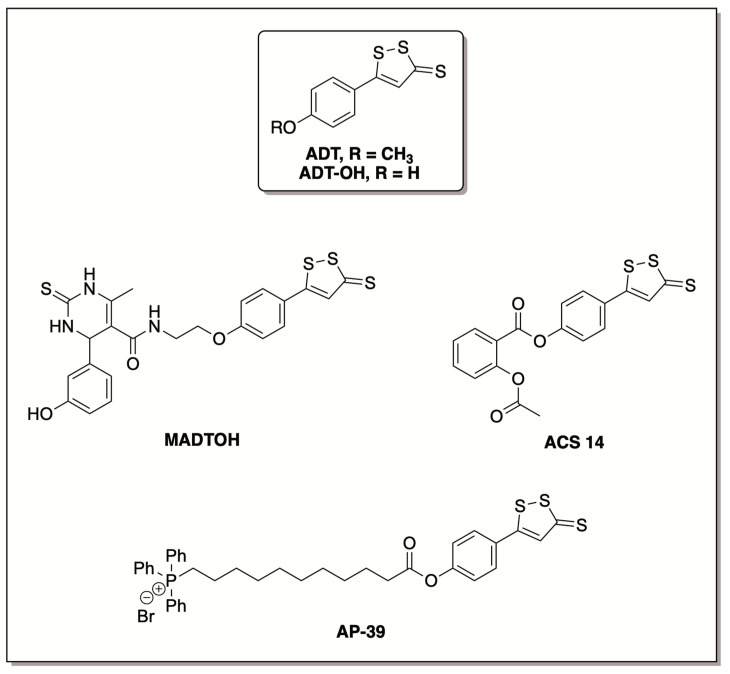
Chemical structures of DTTs and donor hybrids with protective effects against MI/R injury.

ADT and ADT-OH are the most common among this donor class, and their biological properties have been assessed in numerous disease models [86,87,88,89,90,91]. Perhaps most notably, several interesting H_2_S donor hybrids have been obtained by coupling ADT-OH through its phenol onto other therapeutically useful drugs [92], yielding compounds such as MADTOH and ACS14 (Figure 5).

Impressive drug synergism was observed with MADTOH, a monastrol-H_2_S-releasing hybrid, as increased inhibitory effects against L-type calcium channels were observed with this compound in comparison to both monastrol and ADT-OH alone [93]. L-type calcium channel blockers hold promise as an effective therapy for several cardiovascular disorders, including myocardial ischemia [94]. Thus, hybrid molecules, such as MADTOH, may be especially advantageous in treating MI/R injury and warrant further studies.

Along those lines, ACS14 is an H_2_S-releasing, nonsteroidal anti-inflammatory hybrid that combines aspirin and donor ADT-OH. Originally reported on in 2009 [95], ACS14 was first developed in an effort to reduce the gastric toxicity of aspirin by combatting redox imbalance through its release of H_2_S and subsequent increase in heme oxygenase-1 expression. Since this initial report, the cardioprotective effects of ACS14 have also been highlighted in later studies, including its ability to reduce MI/R injury in buthionine sulfoximine-treated rats [96,97].

Similarly, AP39 is an ADT-OH conjugate with impressive therapeutic effects in cardiovascular disease models (Figure 5) [98,99]. By combining ADT-OH with a triphenylphosphonium moiety through an ester linkage, AP39 effectively targets mitochondria, which significantly improves its potency. This was first established in a study aimed at assessing its effects on mitochondrial bioenergetics, which noted that only nanomolar concentrations of AP39 were required to observe stimulatory effects whereas micromolar doses of other H_2_S donors are typically required to evoke similar results. The selective delivery of H_2_S to mitochondria may also heighten its cardioprotective qualities. Indeed, later studies have showcased the ability of AP39 to protect myocardium from ischemia-reperfusion injury by significantly attenuating mitochondrial ROS production and through its stabilization of mitochondrial membrane potentials [100,101,102].

### 4.2. pH-Triggered Donors

**Figure 6:**
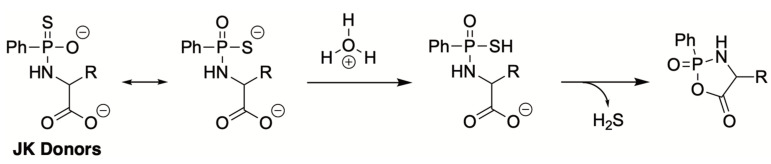
pH-triggered H_2_S release from JK donors.

JK donors are a class of pH-triggered, H_2_S-releasing compounds developed by Xian and co-workers [103]. By appending different amino acids, a series of phosphorothioate-based donors were accessed that undergo an intramolecular cyclization reaction that liberates H_2_S with high efficiency in weakly acidic (pH 5–6) environments (Figure 6). This pathway, however, appears to be inoperable under neutral to slightly basic conditions (pH 7–8), which provides greater spatiotemporal control over their delivery of hydrogen sulfide. These observations are likely to stem from the fact that under weakly acidic conditions, the phosphorothiol moiety is protonated and functions as a good leaving group, while the carboxylate component still resides in its deprotonated, nucleophilic form.

**Figure 7:**
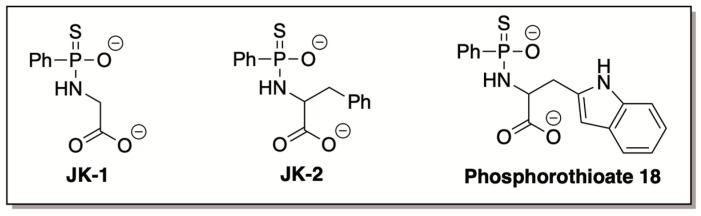
JK donors with established cardioprotective effects in MI/R injury models.

Since numerous pathological conditions are known to lead to a reduction in pH (inflammation, cancer, and cardiovascular disorders), JK donors have the potential to selectively deliver H_2_S under conditions in which a therapeutic benefit is likely to arise. In their original study, the authors successfully demonstrated that both JK-1 and JK-2 (Figure 7) could provide significant cardioprotection in both cellular and in vivo murine models of MI/R injury [103].

It is worth noting that additional donors of this type have been prepared by further modifying the amino acid substituent. Phosphorothioate 18 (Figure 7), for example, was recently accessed and found to protect H9c2 cardiomyocytes from hypoxia-reoxygenation (H/R) injury [104]. In addition, JK-1 was shown to exhibit low toxicity and good pharmacokinetic properties, accentuating the fact that further structure–activity relationship (SAR) studies and additional therapeutic and preclinical profiling within this series is likely to be advantageous.

### 4.3. Thiol-Triggered Donors

**Figure 8:**
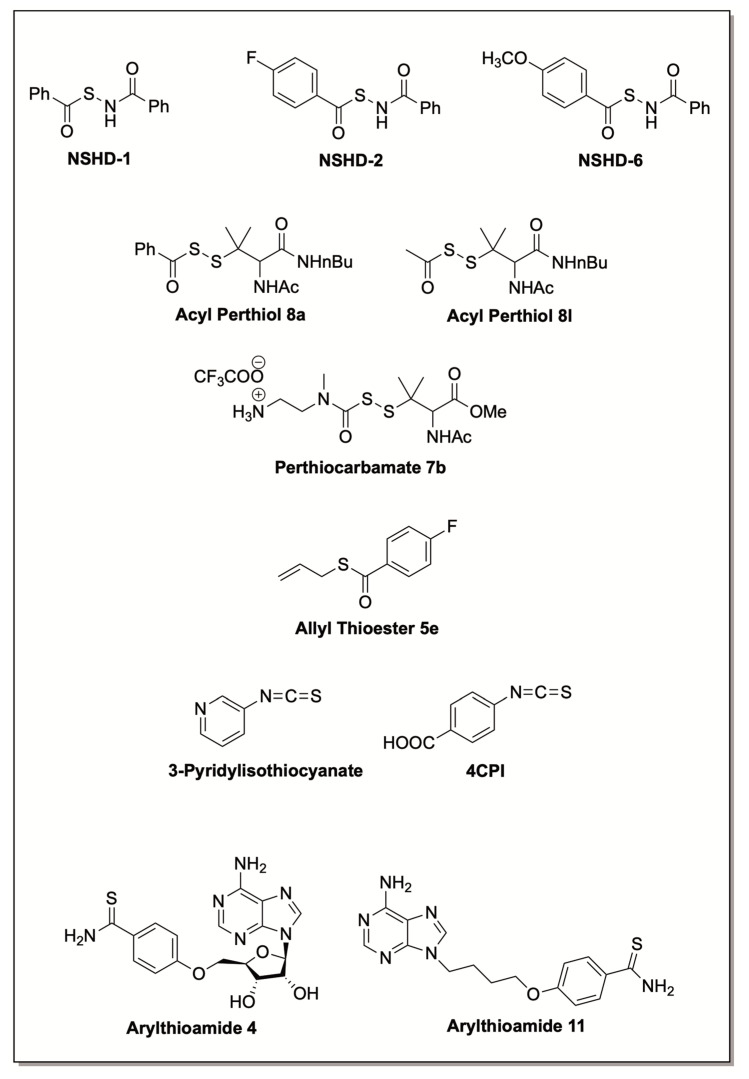
Thiol-triggered donors with established cardioprotective effects in H/R and MI/R injury models.

H_2_S donors selectively responsive to biologically abundant thiols, such as cysteine and glutathione, have also exhibited promising cardioprotective effects. Figure 8 outlines specific compounds within this series that have displayed promising protective effects in MI/R injury models.

Among the first to be examined were a series of *N*-mercapto-based donors (NSHDs) developed by Zhao et al. (Figure 8) [105]. These compounds were shown to be stable in buffer and require the presence of cysteine to effectively deliver H_2_S in aqueous media. Specifically, within this donor class, NSHD-1, NSHD-2, and NSHD-6 demonstrated cytoprotective effects against H_2_O_2_-induced damage in H9c2 cardiomyocytes. Furthermore, NSHD-1 and NSHD-2 also exhibited potent cardioprotective effects in a murine model of MI/R injury.

Additionally, acyl perthiols, allyl thioesters, and perthiocarbamates are responsive to cellular thiols and have established cardioprotective effects in H9c2 cardiomyocytes and other MI/R injury models as a result of their H_2_S release. In the case of acyl perthiols, compounds 8a and 8l demonstrated notable reductions in infarct size relative to vehicle-treated mice in a murine MI/R injury model [58]. Moreover, a significant reduction in circulating cardiac troponin I was observed in both 8a- and 8l-treated mice, which supports the involvement of an H_2_S-related mechanism in their cardioprotection. Within the allyl thioester series, 5e was shown to be the most potent donor in cardiomyocyte (H9c2) models of oxidative damage [106]. It also displayed protective qualities in an in vivo mouse model, reducing infarct size and cardiomyocyte apoptosis. Similarly, perthiocarbamate 7b showcased impressive cardioprotective effects in a Langendorff model of MI/R [107].

Although very electrophilic, isothiocyanates are another class of thiol-activated donors with promising cardioprotective characteristics. In Langendorff-perfused rat hearts, 4CPI was shown to improve post-ischemic recovery through its attenuation of oxidative stress and activation of mitoK_ATP_ channels [108]. Through an extensive SAR study, 3-pyridyl-isothiocynante was identified as another potent donor within this series, exhibiting maximum myocardial protection in an in vivo rat model for acute myocardial infarction at a dose of just 20 µg/kg and from its activation of mitoK_ATP_ channels [109].

Arylthioamides are the final donor class that we will touch upon within this section. What makes arylthioamides distinct from the donors mentioned above is that their release of H_2_S is extremely slow and inefficient, even with the addition of nucleophilic thiols [110]. Moreover, the release of H_2_S from this scaffold proceeds through an unidentified mechanistic pathway. Nevertheless, two hybrid adenine-containing donors, arylthioamide 4 and 11, appear to show synergistic cardioprotective effects by activating the PKG/PLN pathway in ischemic myocardium [111].

Pathways for thiol-triggered release of H_2_S have been explored with these donors. Plausible mechanisms put forth by the authors, based on detailed mechanistic studies, the identification of reaction intermediates, and established organic reactivity, are presented below.

**Figure 9:**
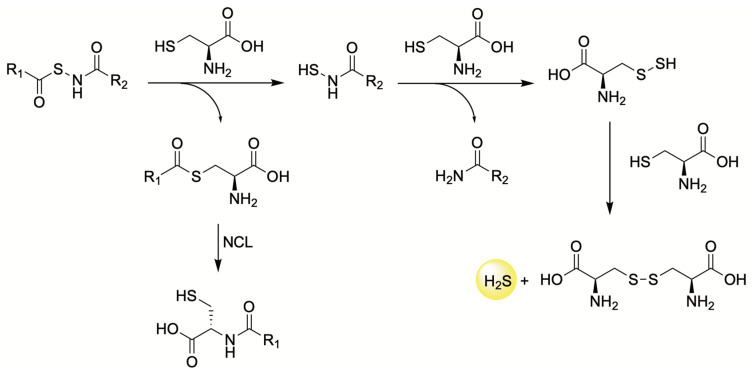
Thiol-triggered H_2_S release from NSHDs.

As depicted in Figure 9, NSHDs initially undergo a nucleophilic acyl substitution with cysteine to form a thioester and an *N*-mercapto (*N*-SH) species. Although this first step is reversible, the ensuing thioester undergoes a rapid *S*-to-*N*-acyl transfer that essentially renders this step irreversible. In the presence of excess cysteine, the *N*-mercapto species is transformed into a primary amide, forming cysteine persulfide in the process. Cysteine persulfide then reacts further with cysteine to generate cystine and free H_2_S. From a detailed SAR analysis, it was discovered that both electronic and steric effects at R_1_ (but not R_2_) influence the rate of H_2_S release [105]. In general, NSHDs with smaller electron-withdrawing substituents at this position exhibited faster kinetics.

**Figure 10:**
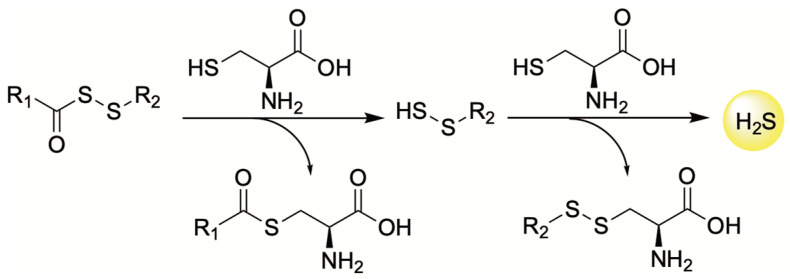
Thiol-triggered H_2_S release from acyl perthiols.

H_2_S can be released from acyl perthiols through an initial thioester exchange reaction that liberates a persulfide [58] (Figure 10). The ensuing hydropersulfide can then undergo an additional thiol exchange reaction to form a disulfide while generating H_2_S.

**Figure 11:**
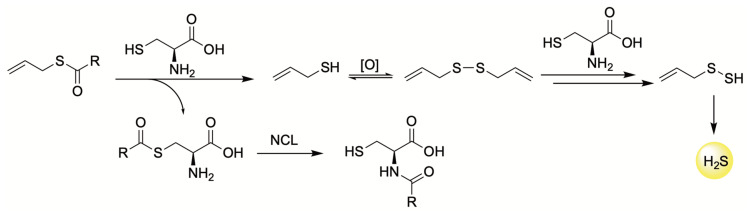
Thiol-triggered H_2_S release from allyl thioesters.

Similarly, allyl thioesters liberate H_2_S by undergoing an initial thioester exchange reaction to generate an allylic thiol, which then oxidizes to form a diallyl disulfide (Figure 11). Diallyl disulfides are known H_2_S donors that are likely to operate through a hydropersulfide intermediate [112,113,114,115,116,117,118].

**Figure 12:**
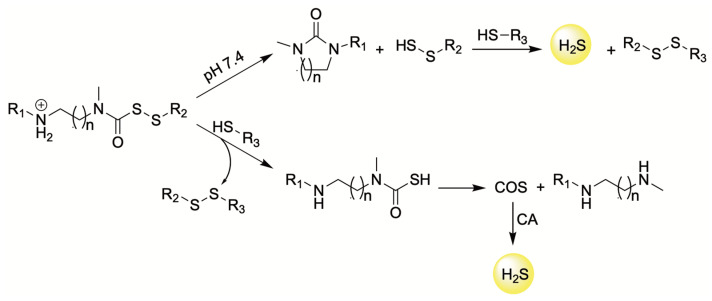
Thiol-triggered H_2_S release from perthiocarbamates.

Within this series of donors, perthiocarbamates are unique in their ability to generate H_2_S from two distinct pathways: hydropersulfide formation and carbonyl sulfide (COS) liberation [107]. As outlined in Figure 12, the COS delivery pathway is initiated by a thiol–disulfide exchange reaction that yields an unstable carbamic thioacid that quickly decomposes and gives rise to COS. In the presence of the ubiquitous enzyme carbonic anhydrase (CA), COS is quickly transformed into H_2_S [119]. Alternatively, perthiocarbamates can liberate H_2_S from a hydropersulfide intermediate that is generated from an intramolecular cyclization reaction that bypasses the need for a specific stimulus to trigger the event.

**Figure 13:**
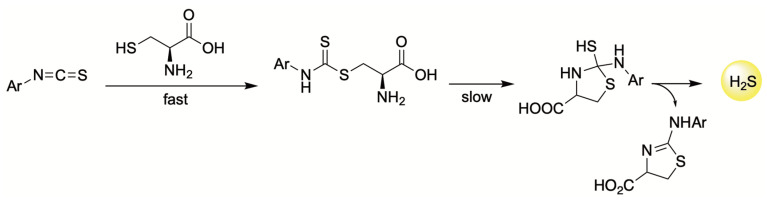
Thiol-triggered H_2_S release from isothiocyanates.

The mechanism of H_2_S liberation from isothiocyanates has been carefully investigated by Lin and co-workers [120]. As delineated in Figure 13, they propose that the reaction commences with the nucleophilic attack by cysteine to form a dithiocarbamate. This intermediate then undergoes an intramolecular cyclization that forms a 5-membered ring and assists in the elimination of H_2_S.

### 4.4. Enzyme-Triggered Donors

Hydrogen sulfide donors that are selectively responsive to specific enzymes have also been developed. Those that have displayed promising protective effects in MI/R injury models are featured below.

**Figure 14:**
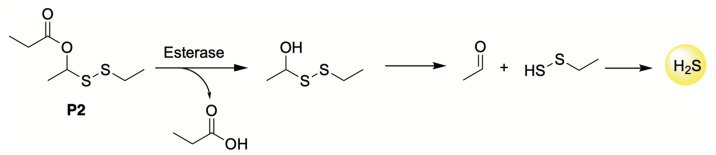
Esterase-triggered H_2_S release from P2, a donor with cardioprotective effects in MI/R injury models.

Esterase enzymes are omnipresent in human cells and, as their name implies, catalyze the hydrolysis of esters [121]. Not surprisingly, H_2_S liberation from a donor that is initiated by esterase-catalyzed hydrolysis is a common approach [122,123,124,125,126,127]. In general, the molecular framework is designed in such a way that upon ester hydrolysis, the resultant alcohol undergoes a self-immolative step that results in the eventual release of H_2_S. Donor P2 (Figure 14) illustrates this approach, as an unstable hydroxymethyl persulfide is unveiled after esterase-catalyzed hydrolysis [128]. This intermediate quickly decomposes to generate acetaldehyde and a hydropersulfide, which serves as an effective H_2_S precursor under biological conditions. Donor P2 was used in a murine model of MI/R injury and displayed promising protective effects with a bell-shaped therapeutic profile.

**Figure 15:**
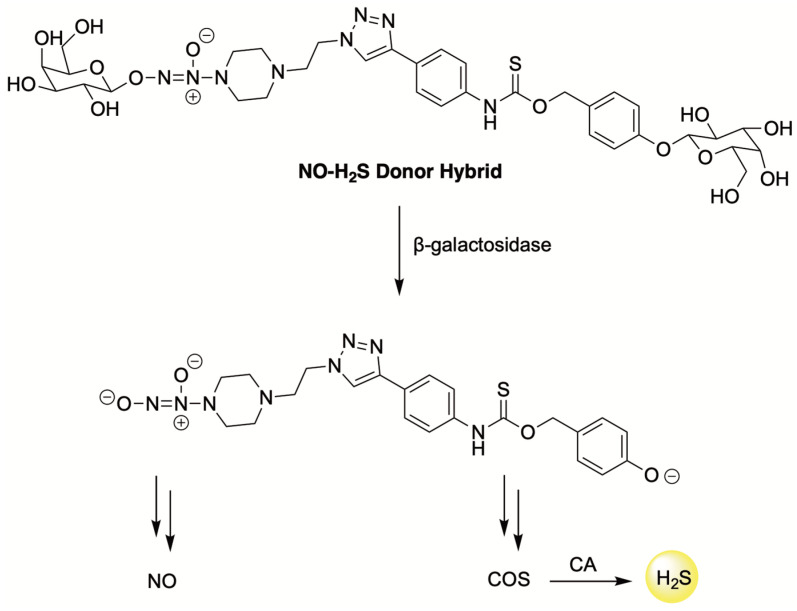
β-galactosidase-triggered H_2_S release from an NO-H_2_S donor hybrid, a compound with cardioprotective effects in MI/R injury models.

Donors selectively responsive to the enzyme β-galactosidase have also displayed favorable cardioprotective effects. The NO-H_2_S donor hybrid depicted in Figure 15 is an example of such a design [129]. In the presence of β-galactosidase, the glycosidic bonds in the molecule are cleaved, producing an unstable intermediate that further unravels to liberate H_2_S (via COS hydrolysis) and nitric oxide (NO). To underline its cardioprotective effects, this hybrid prodrug was used in a rat model of heart failure. In general, it was shown that administration of the NO-H_2_S donor hybrid noticeably improved cardiac function post myocardial infarction, and especially in comparison to NO or H_2_S treatment alone, highlighting the effectiveness of a hybrid approach.

### 4.5. ROS-Triggered Donors

**Figure 16:**
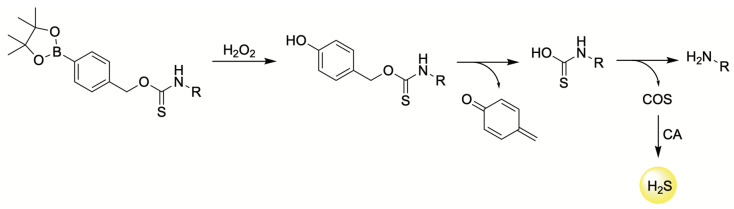
General mechanism for H_2_S release from ROS-triggered, *O*-alkyl thiocarbamate-based donors.

H_2_S donors selectively responsive to elevated levels of ROS have been shown to be especially advantageous at combatting oxidative stress-related diseases [130,131,132,133,134], including MI/R injury. Within the structural framework of these donors, an *O*- or *S*-alkyl thiocarbamate is often linked to an aryl boronate ester, which serves as an ROS-responsive trigger [135,136,137]. In the presence of ROS (especially hydrogen peroxide or peroxynitrite), the aryl boronate ester is quickly oxidized to an unstable phenol that undergoes a 1,6-elimination to provide H_2_S through carbonic anhydrase catalyzed COS hydrolysis (Figure 16).

**Figure 17:**
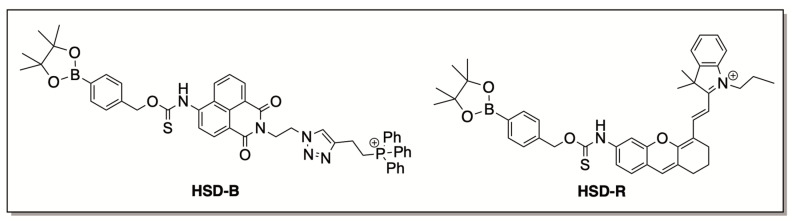
ROS-triggered donors with cardioprotective effects in H/R and MI/R injury models.

An advantage of donors that proceed through the COS/H_2_S pathway is their concurrent release of an aryl amine (or aryl alcohol) which affords an easy opportunity to access self-reporting donors that can track their H_2_S delivery via fluorescence spectroscopy and other imaging techniques [134,138,139,140]. HSD-B and HSD-R (Figure 17) serve as examples of this, due to there being a latent fluorescent reporter embedded within their *O*-alkyl thiocarbamate framework. Moreover, these compounds were rationally designed to target mitochondria, thanks to their lipophilicity and cationic charge, which are likely to contribute to their pronounced cardioprotective effects that have been observed in H/R injury models [139,140]. HSD-B, for example, was shown to provide protection in a H9c2 cellular model of H/R injury, while HSD-R exhibited anti-apoptotic (inhibition of pro-apoptotic genes, including Bid, Apaf-1, and P53), anti-inflammatory, and pro-angiogenic effects in a rat MI/R injury model.

## 5. Chemotherapy-Induced Cardiotoxicity

Chemotherapy-induced cardiotoxicity is a serious complication that affects the long-term survival of cancer patients and often manifests itself several years to several decades after the completion of treatment [141,142]. By convention, chemotherapy-induced cardiotoxicity is sorted into two distinct categories: type I, which is more severe, dose-dependent, and triggered by anthracycline-based drugs [143,144,145], and type II, which is less severe, believed to be reversible upon the cessation of treatment, and associated with cisplatin, alkylating agents, antimetabolites, and other non-anthracycline-based chemotherapeutics [146].

Anthracyclines, such as doxorubicin (DOX) and daunorubicin, are among the most effective anticancer agents in clinical use [147]. Their planar anthraquinone tetracyclic structure allows them to insert between DNA base pairs and interfere with the enzyme topoisomerase II, which, in turn, prevents the DNA unwinding and replication that ultimately induces apoptosis in proliferating cancer cells [148]. However, the same chemical features that give rise to DNA intercalation also predispose anthracyclines to redox cycling that generates superfluous levels of ROS within a cellular environment and, specifically, in mitochondria [149,150]. With increased mitochondrial density and a relatively deficient antioxidant defense system in place, cardiomyocytes are especially susceptible to oxidative injury [151,152]. Therefore, while other mechanisms may be in play, the uncontrolled production of ROS is believed to be primarily responsible for the dose-dependent, irreversible heart damage that is observed with anthracycline-based chemotherapeutics [153,154,155].

**Figure 18:**
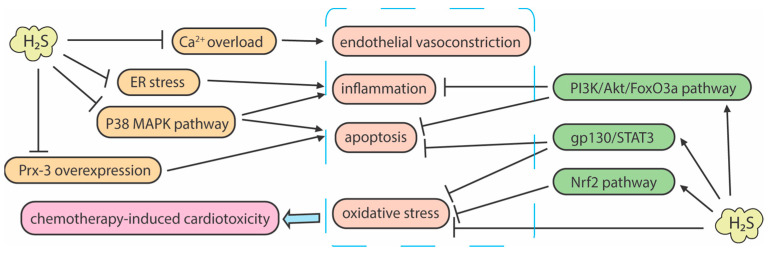
Protective mechanisms of H_2_S against chemotherapy-induced cardiotoxicity.

Given the significance of anthracyclines in the fight against cancer, it comes as no surprise that new therapeutic strategies are being extensively explored in an effort to diminish their cardiotoxic side effects. To this end, it has been suggested that the co-administration of H_2_S—with its impressive antioxidative, anti-inflammatory, and anti-apoptotic effects—may offer an effective solution [156]. This hypothesis was first explored by Su and co-workers in 2009, using a DOX-treated rat model [157]. Employing NaHS as an H_2_S donor, the attenuation of DOX-induced mitochondrial injury was in fact observed, along with significant improvements in overall cardiac function. Subsequent investigations have corroborated these initial findings, and the beneficial effects of H_2_S are now well-established for combatting chemotherapy-induced cardiotoxicity of both type 1 and type 2 through various mechanisms (Figure 18) [158,159,160,161,162].

While the co-administration of H_2_S appears to be a promising approach for reducing the cardiotoxic profile of drug molecules, efforts to improve absorption and target delivery have led to the emergence of a new codrug design in which a known H_2_S-donating moiety is directly linked to a chemotherapeutic agent of interest. This strategy is akin to the ABT-OH donor hybrids discussed earlier (Section 4.1) and has proven to be especially beneficial for mitigating DOX-induced cardiotoxicity, in particular. Therefore, given their obvious translational potential and clever chemical design, these hybrid DOX molecules are detailed below.

## 6. H_2_S Conjugated Codrugs That Combat Anthracycline-Induced Cardiotoxicity

**Figure 19:**
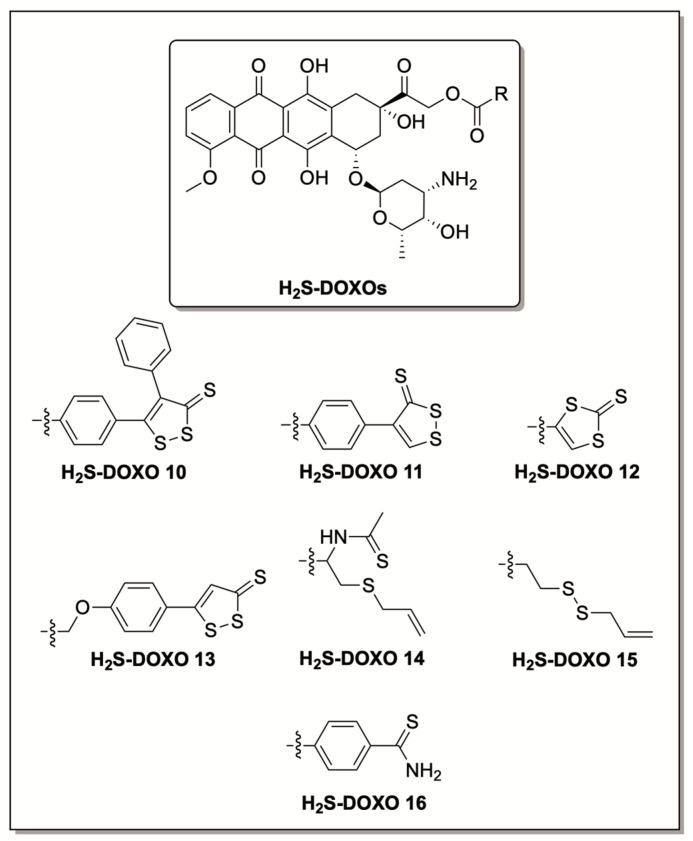
H_2_S-donating, doxorubicin hybrid codrugs with protective effects against anthracycline-induced cardiotoxicity.

Chegaev and co-workers were the first to synthesize and assess a series of H_2_S-releasing, DOX hybrid codrugs (termed H_2_S-DOXOs) [163]. To accomplish this, they appended known H_2_S-donating motifs via an ester bond at C-14 of DOX. As seen in Figure 19, the affixed H_2_S-donating moieties included DTT derivative (H_2_S-DOXOs 10–13), allyl sulfide (H_2_S-DOXO 14), allyl disulfide (H_2_S-DOXO 15), and an aryl thioamide (H_2_S-DOXO 16).

After verifying H_2_S liberation from H_2_S-DOXOs in cell culture media, an LDH assay was used to assess their cytotoxic effects in H9c2 cardiomyocytes in culture. Compared to DOX, H_2_S-DOXOs 10–14 were found to be significantly less cytotoxic, and the addition of the H_2_S scavenger hydroxocobalamin confirmed that their release of hydrogen sulfide was responsible for their reduced cardiotoxicity.

Perhaps most notably, however, H_2_S-DOXOs 10 and 11 simultaneously displayed impressive anticancer activity in human osteosarcoma cells (U-20S), even compared to the parent drug. Follow-up studies with H_2_S-DOXO 10 indicated that the increased potency is likely to stem from their disruption of drug efflux by Pgp [164], which increases their cellular concentration. Thus, the appendage of an H_2_S donor to DOX appears to impart several distinct advantages, including improved functional activity against multidrug-resistant cancers in addition to a reduced cardiotoxic profile.

Since this initial study, H_2_S-DOXO 10 (or Sdox) has undergone additional preclinical studies (Table 2) [165,166,167]. In a DOX-resistant prostate cancer mouse model, treatment with Sdox led to significantly reduced tumor volumes and improved safety. Conversely, DOX-treated mice exhibited reduced body weight and cardiotoxicity, which was assessed by measuring troponin plasma levels and left-ventricular-wall thickness.

**Figure 20:**
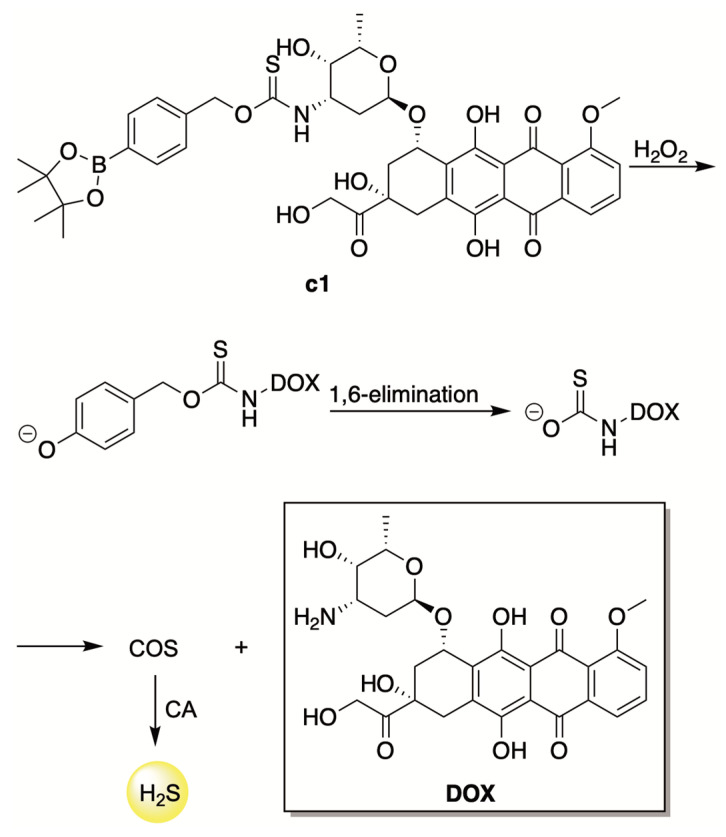
ROS-triggered H_2_S release from c1, an ROS-responsive DOX hybrid prodrug with reduced cardiotoxicity in rat cardiomyocytes in culture.

In a similar fashion, Hu et al. recently reported on an H_2_S-releasing, DOX hybrid codrug (c1, Figure 20) [168]. However, unlike H_2_S-DOXOs, c1 is a prodrug that only liberates active DOX and H_2_S under conditions of oxidative stress.

Elevated levels of ROS are found in most cancers for a variety of reasons [169]. Consequently, ROS-inducible anticancer prodrugs have emerged as a promising design strategy for improving the therapeutic index of anticancer chemotherapeutic agents [170,171,172,173]. Thus, the design of c1 represents a novel strategy that imparts both tumor-selective activation and H_2_S delivery in combination to further reduce the cardiotoxic side effects of DOX.

As highlighted in Figure 20, c1 utilizes an aryl boronate ester as an H_2_O_2_-selective trigger. Upon its oxidation by peroxide, the ensuing phenol undergoes a 1,6-elimination that releases both H_2_S (by way of COS hydrolysis) and DOX. The authors confirmed this mechanism through LCMS studies and verified the selective release of both DOX and H_2_S in response to H_2_O_2_.

The toxicity of c1 was assessed in rat cardiomyocytes in culture (Table 2). Using this model, c1 exhibited reduced cardiotoxicity compared to that of DOX. By enlisting an H_2_O_2_-activated DOX prodrug as a control, which provided CO_2_ release rather than COS, it was concluded that the protective effects of c1 are likely to stem from its co-release of H_2_S. Cells treated with c1 also evinced significantly higher Nrf2 activation and heme oxygenase-1 expression compared to controls, providing a likely mechanism of cellular protection [174,175,176,177].

Notably, c1 also appeared to maintain the antitumor effects of DOX in a 4T1 mouse breast-cancer cell line. Therefore, while further preclinical profiling—especially in vivo—is required, the selective tumor activation and H_2_S liberation provided by c1 offer further promising options for overcoming DOX-derived cardiotoxicity in the clinic.

## 7. Conclusions

Once regarded as merely a toxic and foul-smelling gas, H_2_S has more recently been recognized as a key signaling molecule and important endogenous mediator of numerous physiological and pathophysiological processes within mammalian systems. Its positive influence on the cardiovascular system, in particular, is rooted in its involvement in vasodilation (activation of K_ATP_ channels and the PI3K/Akt signaling pathway) [178,179,180,181], as well as its anti-inflammatory (inhibition of the p38 MAPK/NF-κB pathway) [182,183], antioxidative (activation of the Nrf2 signaling pathway) [59,168,184], and anti-apoptotic (suppression of pro-apoptotic genes Bid, Apaf-1, and p53) [140] properties, which have been extensively reviewed elsewhere in the literature [54,55,116,185].

Exogenous supplementation with H_2_S has been shown to vastly improve outcomes in various in vitro and in vivo cardiovascular disease models. In this review, its effectiveness at combating MI/R injury and chemotherapy-induced cardiotoxicity was explored, along with the fundamental chemistry and H_2_S releasing mechanism of the donor molecules that were utilized in these studies. The continued development and refinement of H_2_S-releasing compounds is critical to unlocking the translational therapeutic potential of hydrogen sulfide, by augmenting its delivery and bioavailability while better mimicking its natural and prolonged enzymatic production. Thus, the compounds reported on herein not only represent important investigative tools for probing the chemical biology of hydrogen sulfide but may also one day serve as important therapeutic agents for the treatment of MI/R injury and anthracycline-induced cardiotoxicity.

## Figures and Tables

**Table 1:** Synthetic H_2_S donors with documented protective effects against MI/R injury.

H_2_S Donor	Release Mechanism	Preclinical Studies
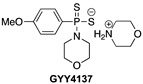	Hydrolysis-Triggered	In vivo rat and diabetic mice models of MI/R injury
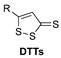	Hydrolysis-Triggered	Rat isolated perfused heart model of MI/R injury
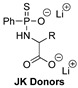	pH-Triggered	H9c2 cardiomyocyte model of H/R injury and in vivo murine model of MI/R injury
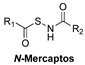	Thiol-Triggered	In vivo murine model of MI/R injury
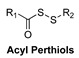	Thiol-Triggered	In vivo murine model of MI/R injury
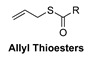	Thiol-Triggered	In vivo murine model of MI/R injury
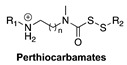	Thiol-Triggered	Mouse isolated perfused heart model of MI/R injury
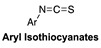	Thiol-Triggered	Rat isolated perfused heart model and in vivo murine model of MI/R Injury
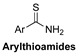	Thiol-Triggered	In vivo rabbit model of MI/R injury
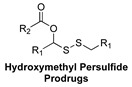	Enzyme-Triggered	in vivo murine model of MI/R injury
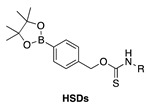	ROS-Triggered	H9c2 cardiomyocyte model of H/R injury and in vivo murine model of MI/R injury

**Table 2:** H_2_S-releasing hybrid codrugs with protective effects against anthracycline-induced cardiotoxicity.

H2S Hybrid	Release Mechanism	Preclinical Studies
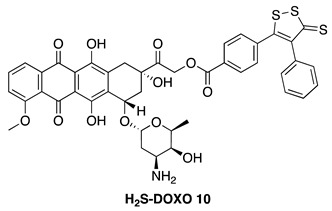	Hydrolysis-Triggered	H9c2 cardiomyocytes, U-2OS osteosarcoma cells, DU-145 prostate cancer cells, and in vivo DOX-resistant mouse models
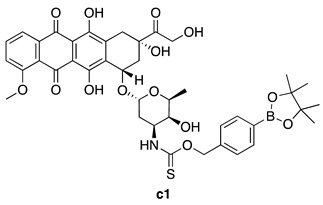	ROS-Triggered	H9c2 cardiomyocytes and 4T1 breast cancer cells
